# Heart to heart: grafting cardiosphere-derived cells augments cardiac self-repair by both myocytes and stem cells

**DOI:** 10.1002/emmm.201202345

**Published:** 2013-02-04

**Authors:** Jose A Palacios, Michael D Schneider

**Affiliations:** British Heart Foundation Centre of Research Excellence, National Heart and Lung Institute, Imperial College LondonLondon, UK

**Keywords:** cardiac regeneration, cell therapy, myocardial infarction, regeneration, stem cells

→See related article in EMBO Molecular Medicine http://dx.doi.org/10.1002/emmm.201201737

Compared with highly regenerative organisms such as newts and zebrafish (Kikuchi & Poss, 2012), the capacity of mammalian adult heart tissue to undergo self-repair is insufficient to reconstitute the muscle lost in myocardial infarction, hindering functional recovery from heart attacks and from cell loss occurring in chronic heart failure (Mercola et al, 2011). The irreversibility of cell cycle exit in adult cardiomyocytes largely prevents the restoration of pump function via proliferation of pre-formed myocytes. However, overriding or inactivating tumour suppressor pocket proteins is one route to engineer persistent cycling in the adult heart (Mercola et al, 2011), and low levels of on-going myocyte generation have been demonstrated by fate-mapping in mice (Hsieh et al, 2007) and ^14^C dating in human heart tissue (Bergmann et al, 2009). Although quite limited, at least in healthy aging hearts, such turnover is conceptually important, as the underlying mechanism(s) could potentially be exploited by the body itself or the clinician after injury.

Where do such myocytes come from, and what is the extent of cardiomyocyte generation in disease? A highly plausible source is suggested by the discovery of dormant or latent cells with cardiogenic potential in adult hearts (Mercola et al, 2011). Purified by a variety of means, these cells have in common the expression of many heart-forming transcription factors, and encouraging phase I trials have been reported using them as autologous cell products (SCIPIO, CADUCEUS; Bolli et al, 2011; Makkar et al, 2012). Alternatively, the robust scarless healing of the heart in zebrafish occurs by differentiated myocytes re-entering the cell cycle (Kikuchi & Poss, 2012), a mechanism that is available to mice only during the first days of life (Porrello et al, 2011). These findings have already spurred a renaissance of cardiac cell cycle studies, seeking safe, controllable means to restart proliferation in adult cardiomyocytes (Eulalio et al, 2012). But is it the plasticity of cycling or the plasticity of differentiation that gives rise to new cardiomyocytes in injured hearts?

The potential translational importance of these two related, but distinguishable pathways is heightened by knowledge from cell grafting for cardiac repair, namely that bone marrow cells likely do not trans-differentiate, and even heart-derived cells do not durably engraft (Mercola et al, 2011). Consequently, for both of the cell types in widest use in human trials, paracrine effects on self-repair are under scrutiny.

Among the strongest estimates of myocyte generation are the findings from fate-mapping—irreversibly tagging the cardiomyocytes that exist prior to injury with lineage-restricted (Myh6-driven) tamoxifen-dependent Cre recombinase, thereby excising one reporter gene (LacZ) while activating a second (green fluorescent protein; GFP), during a brief temporal window. In this form of “pulse-chase” design, dilution of the initially LacZ^−^GFP^+^ cardiomyocytes by LacZ^+^GFP^−^ cells signifies cardiomyocyte replacement from a starting non-myocyte population (stem cells or other undifferentiated progenitors). By this technique, little cell turnover was seen with normal aging in mice, whereas roughly 15% of the cardiomyocytes in injured myocardium were derived from non-myocyte precursors (Hsieh et al, 2007). Moreover, the quantitative replacement of GFP by LacZ-expressing myocytes excludes factitious dilution of the starting myocytes, as might occur by reductions in transgene expression via DNA methylation and transcriptional silencing. An equally exciting discovery made using this system was the evidence that the beneficial effect of c-kit^+^ bone marrow cells on the injured mouse heart involved myocyte generation by the activation of endogenous cardiac progenitor or stem cells (Loffredo et al, 2011). This effect was not seen with bone marrow mesenchymal stem cells, excluding a non-descript or generic effect of cell delivery and raising the possibility that other cell types might be even more active.

»…these findings suggest a contribution to new myocyte formation following infarction both from pre-existing myocytes and from undifferentiated precursor cells«

In this issue of EMBO Molecular Medicine, Malliaras and colleagues have applied this lineage tracing system, together with other tools, to address the thematically related question of how heart-derived cells benefit the heart (Malliaras et al, 2013). Using mouse cardiosphere-derived cells that correspond to the human cells tested in CADUCEUS (Makkar et al, 2012), one striking effect of cell grafting was an increase in cycling of pre-existing myocytes, as measured by Ki67, BrdU incorporation into DNA, and histone H3 phospho-epitope staining, which denotes Cdc2 activity in mitosis. Cycling myocytes tended to be small, mono-nucleated, and adjacent to the area of injury. In addition, the authors notably observed the dilution of GFP^+^ myocytes by GFP^−^ ones, and an even larger increase in cycling of this GFP^−^ cardiomyocyte population. Together, these findings suggest a contribution to new myocyte formation following infarction both from pre-existing myocytes and from undifferentiated precursor cells, with enhancement of both regenerative pathways by cell grafting (Fig 1). Numerous precautions were undertaken to exclude alternative explanations like DNA synthesis without karyokinesis and cytokinesis. For instance, roughly 10% of the BrdU^+^ cardiomyocyte nuclei were more than diploid, but this small fraction would not account for the observed extent of cell cycle activity. Few grafted cells survived beyond the first three weeks, signifying that the observed benefits in cardiomyocyte number, cardiac geometry, and cardiac pump function should be ascribed to enhancing self-repair, not an enduring role of the grafted cells themselves. Differences from earlier fate-mapping studies that failed to detect a similar contribution by preformed cardiomyocytes (Hsieh et al, 2007; Loffredo et al, 2011) were viewed as likely arising from the greater sensitivity here, using dissociated cells plus longer BrdU pulses. In addition, paracrine differences between heart-derived cells and bone marrow cells cannot be excluded as a basis for different outcomes between the modes of cell therapy tested.

**Figure 1:**
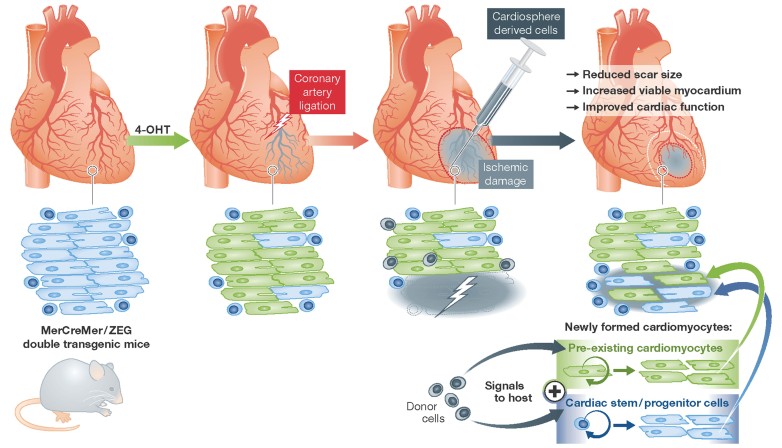
Cardiosphere-derived cells stimulate the formation of new cardiomyocytes following infarction both from preformed cardiomyocytes and from undifferentiated progenitor or stem cells Donor cells did not stably engraft, yet conferred an improvement in cardiac structure and pump function. Fate-mapping with the Cre/lox system ascribed new cardiomyocyte creation both to proliferation of pre-existing myocytes and to the differentiation of formerly undifferentiated cells.

An intriguing article in Nature by the group that pioneered fate-mapping in cardiac self-repair (Hsieh et al, 2007; Loffredo et al, 2011) has re-examined this vexing question about the cellular origin of newly formed cardiomyocytes after myocardial infarction (Senyo et al, 2012). Their Cre/lox system was employed again, as also was used by Malliaras and colleagues, but with the technical refinement that DNA replication was assessed by the incorporation of ^15^N thymidine, a stable isotope, using high-resolution imaging mass spectrometry. The combination of these two pulse-chase techniques—GFP fluorescence and ^15^N/^14^N ratiometric imaging—allowed the researchers to compare the extent of DNA replication after injury in cardiomyocytes derived from pre-existing ones *versus* cardiomyocytes derived from undifferentiated precursors. With this change in methodology, Senyo and colleagues drew conclusions contrasting with their previously reported results: the prevalence of ^15^N labelling did not differ significantly between GFP^+^ and GFP^−^ myocytes, suggesting that dilution of the GFP^+^ cardiomyocyte pool cannot be explained by expansion and differentiation of endogenous stem or progenitor cells.

Both studies concur that newly generated cardiomyocytes are formed from pre-existing ones. The intriguing discrepancy concerns whether undifferentiated cardiac precursors contribute to the formation of new heart muscle after injury. Why these discordant results? Inherently, any number of technical differences could influence the observed results, although the mouse strain, age and injury models were comparable.

One matter is sample size. Multi-isotope imaging mass spectrometry permits unique and unsurpassably detailed information, but necessarily on a far smaller scale than accessible to flow cytometry. Given that the total number of ^15^N^+^ cardiomyocytes was just 35 (out of more than 4000 sampled), this number might simply be too small to clearly resolve potential differences in prevalence between GFP^+^ and GFP^−^ myocyte subpopulations. Also, since genetic recombination triggered by tamoxifen is incomplete even after thorough optimization, 80% of pre-formed myocytes are GFP^+^, but 20% are already GFP^−^ and the potential contribution of myocytes from a stem cell pool is at best likely confined to the 10% increase in GFP^−^ cardiomyocytes. The mass spectrometry study can exclude stem cells as the sole source of new myocytes, but not necessarily as one source among others. In addition, the exact timing and extent of endogeneous stem cell expansion and differentiation are still unsettled. A second matter, therefore, is the need for improved genetic tools to track the fate of cardiac stem cells more directly than by extrapolation from dilution. Such an approach has been instrumental, for example, in pinpointing the contribution of new myocytes from epicardial stem cells following infarction and treatment with the protein thymosin β4 (Smart et al, 2011).

In summary, Malliaras and colleagues provide important new findings on the underlying cellular basis for improvements in cardiac structure and function from cardiosphere-derived cells, as observed in experimental animals and the ground-breaking phase 1 CADUCEUS trial. The results implicate self-repair as the principal target of these heart-derived cells in the treatment of heart disease, until persistent engraftment of mechanically active and well-integrated cardiomyocytes is achieved. Further research will be necessary to resolve uncertainties about the comparative role of progenitor cells and cardiomyocytes in the formation of new heart muscle after injury. Even cardiomyocyte proliferation remains a subject of dispute, with further new tools now entering the armamentarium (Hesse et al, 2012).

## References

[b1] Bergmann O, Bhardwaj RD, Bernard S, Zdunek S, Barnabe-Heider F, Walsh S, Zupicich J, Alkass K, Buchholz BA, Druid H (2009). Evidence for cardiomyocyte renewal in humans. Science.

[b2] Bolli R, Chugh AR, D'Amario D, Loughran JH, Stoddard MF, Ikram S, Beache GM, Wagner SG, Leri A, Hosoda T (2011). Cardiac stem cells in patients with ischaemic cardiomyopathy (SCIPIO): initial results of a randomised phase 1 trial. Lancet.

[b3] Eulalio A, Mano M, Ferro MD, Zentilin L, Sinagra G, Zacchigna S, Giacca M (2012). Functional screening identifies miRNAs inducing cardiac regeneration. Nature.

[b4] Hesse M, Raulf A, Pilz GA, Haberlandt C, Klein AM, Jabs R, Zaehres H, Fugemann CJ, Zimmermann K, Trebicka J (2012). Direct visualization of cell division using high-resolution imaging of M-phase of the cell cycle. Nat Commun.

[b5] Hsieh PC, Segers VF, Davis ME, MacGillivray C, Gannon J, Molkentin JD, Robbins J, Lee RT (2007). Evidence from a genetic fate-mapping study that stem cells refresh adult mammalian cardiomyocytes after injury. Nat Med.

[b6] Kikuchi K, Poss KD (2012). Cardiac regenerative capacity and mechanisms. Annu Rev Cell Dev Biol.

[b7] Loffredo FS, Steinhauser ML, Gannon J, Lee RT (2011). Bone marrow-derived cell therapy stimulates endogenous cardiomyocyte progenitors and promotes cardiac repair. Cell Stem Cell.

[b8] Makkar RR, Smith RR, Cheng K, Malliaras K, Thomson LEJ, Berman D, Czer LSC, Marban L, Mendizabal A, Johnston PV (2012). Intracoronary cardiosphere-derived cells for heart regeneration after myocardial infarction (CADUCEUS): a prospective, randomised phase 1 trial. Lancet.

[b9] Malliaras K, Zhang Y, Seinfeld J, Galang G, Tseliou E, Cheng K, Sun B, Aminzadeh M, Marbán E (2013). Cardiomyocyte proliferation and progenitor cell recruitment underlie therapeutic regeneration after myocardial infarction in the adult mouse heart. EMBO Mol Med.

[b10] Mercola M, Ruiz-Lozano P, Schneider MD (2011). Cardiac muscle regeneration: lessons from development. Genes Dev.

[b11] Porrello ER, Mahmoud AI, Simpson E, Hill JA, Richardson JA, Olson EN, Sadek HA (2011). Transient regenerative potential of the neonatal mouse heart. Science.

[b12] Senyo SE, Steinhauser ML, Pizzimenti CL, Yang VK, Cai L, Wang M, Wu TD, Guerquin-Kern JL, Lechene CP, Lee RT (2012). Mammalian heart renewal by pre-existing cardiomyocytes. Nature.

[b13] Smart N, Bollini S, Dube KN, Vieira JM, Zhou B, Davidson S, Yellon D, Riegler J, Price AN, Lythgoe MF (2011). De novo cardiomyocytes from within the activated adult heart after injury. Nature.

